# Which patients benefit from adding short-term psychodynamic psychotherapy to antidepressants in the treatment of depression? A systematic review and meta-analysis of individual participant data

**DOI:** 10.1017/S0033291722003270

**Published:** 2023-10

**Authors:** Ellen Driessen, Marjolein Fokkema, Jack J. M. Dekker, Jaap Peen, Henricus L. Van, Giuseppe Maina, Gianluca Rosso, Sylvia Rigardetto, Francesco Cuniberti, Veronica G. Vitriol, Antonio Andreoli, Yvonne Burnand, Jaime López Rodríguez, Valerio Villamil Salcedo, Jos W. R. Twisk, Frederik J. Wienicke, Pim Cuijpers

**Affiliations:** 1 Department of Clinical Psychology, Behavioural Science Institute, Radboud University, Nijmegen, Netherlands; 2 Depression Expertise Centre, Pro Persona Mental Health Care, Nijmegen, Netherlands; 3 Department of Clinical, Neuro and Developmental Psychology, Amsterdam Public Health Research Institute, Vrije Universiteit Amsterdam, Amsterdam, Netherlands; 4 Department of Methodology and Statistics, Leiden University, Leiden, Netherlands; 5 Department of Research, Arkin Mental Health Care, Amsterdam, Netherlands; 6 NPI, Arkin Mental Health Care, Amsterdam, Netherlands; 7 Department of Neuroscience ‘Rita Levi Montalcini’, University of Turin, Turin, Italy; 8 Psychiatric Unit, San Luigi Gonzaga University Hospital of Orbassano, Turin, Italy; 9 Medical School, University of Talca, Talca, Chile; 10 Geneva University Hospital Center, Geneva, Switzerland; 11 National Institute of Psychiatry, Mexico City, Mexico; 12 Department of Epidemiology and Data Science, Amsterdam University Medical Centers, Amsterdam, Netherlands

**Keywords:** Combined treatment, depression, individual participant data meta-analysis, moderator, short-term psychodynamic psychotherapy (STPP)

## Abstract

**Background:**

Adding short-term psychodynamic psychotherapy (STPP) to antidepressants increases treatment efficacy, but it is unclear which patients benefit specifically. This study examined efficacy moderators of combined treatment (STPP + antidepressants) *v.* antidepressants for adults with depression.

**Methods:**

For this systematic review and meta-analysis (PROSPERO registration number: CRD42017056029), we searched PubMed, PsycINFO, Embase.com, and the Cochrane Library from inception to 1 January 2022. We included randomized clinical trials comparing combined treatment (antidepressants + individual outpatient STPP) *v.* antidepressants in the acute-phase treatment of depression in adults. Individual participant data were requested and analyzed combinedly using mixed-effects models (adding Cochrane risk of bias items as covariates) and an exploratory machine learning technique. The primary outcome was post-treatment depression symptom level.

**Results:**

Data were obtained for all seven trials identified (100%, *n* = 482, combined: *n* = 238, antidepressants: *n* = 244). Adding STPP to antidepressants was more efficacious for patients with high rather than low baseline depression levels [*B* = −0.49, 95% confidence interval (CI) −0.61 to −0.37, *p* < 0.0001] and for patients with a depressive episode duration of >2 years rather than <1 year (*B* = −0.68, 95% CI −1.31 to −0.05, *p* = 0.03) and than 1–2 years (*B* = −0.86, 95% CI −1.66 to −0.06, *p* = 0.04). Heterogeneity was low. Effects were replicated in analyses controlling for risk of bias.

**Conclusions:**

To our knowledge, this is the first study that examines moderators across trials assessing the addition of STPP to antidepressants. These findings need validation but suggest that depression severity and episode duration are factors to consider when adding STPP to antidepressants and might contribute to personalizing treatment selection for depression.

Short-term psychodynamic psychotherapy (STPP) is an empirically supported treatment for depression (Driessen et al., 2015) that is frequently applied in clinical practice. Reviews have concluded that combined treatment of antidepressants and STPP is more efficacious than antidepressants alone (Driessen et al., 2020; Fonagy, 2015). However, this effect – on average – is small at treatment completion (Driessen et al., 2020) and it is unclear which patients benefit specifically. Due to scarcity of treatment resources and because adding STPP to antidepressants requires a financial investment, it is of considerable clinical importance to know for whom the addition is beneficial and for whom this might not be the case.

A main reason why it is unclear which pre-treatment patient characteristics (so-called moderators) are associated with differential response to combined treatment of antidepressants and STPP, is lack of statistical power in individual clinical trials, which have sample sizes aimed at identifying intervention effects rather than moderators. Similarly, ‘conventional’ meta-analyses, which are based on study-level characteristics extracted from published trial reports, have not been able to examine moderators in this regard because the number of available studies was too small (Driessen et al., 2015). Moreover, such analyses would have been prone to ecological bias, such that the association between the study-level characteristics may not be representative of the true relationships in the data at the individual level.

‘Individual participant data’ (IPD) meta-analysis is an alternative technique to examine treatment effects by combining patient-level data from multiple clinical trials, which increases the statistical power to examine moderators of treatment efficacy (Lambert, Sutton, Abrams, & Jones, 2002). Furthermore, because moderators are studied at the patient level, ecological bias can be circumvented. We, therefore, conducted a systematic review and IPD meta-analysis to examine efficacy moderators of acute-phase combined treatment (antidepressants + STPP) *v.* antidepressants [with/without brief supportive psychotherapy (BSP)] as compared on depressive symptom measures in randomized clinical trials for adults with depression.

## Methods

### Design

This study is part of a systematic review and IPD meta-analysis project aimed at examining different aspects of STPP for depression efficacy. This larger project was registered (PROSPERO registration number: CRD42017056029) and its study protocol was published (Driessen et al., 2018). This report complies with the Preferred Reporting Items for Systematic Review and Meta-Analyses of Individual Participant Data (PRISMA-IPD) statement (Stewart et al., 2015).

### Study selection

We searched five bibliographic databases (PubMed, PsycINFO, Embase.com, Web of Science, and Cochrane's Central Register of Controlled Trials), two gray-literature databases (GLIN, UMI ProQuest), and a prospective trial register (http://www.controlled-trials.com) from inception to 19 June 2017, without applying language or date restrictions (for the full electronic search strategy see Driessen et al., 2018). We also searched references of psychodynamic therapy meta-analyses and contacted psychodynamic therapy researchers. Finally, in order to identify recent studies, we searched a database of randomized depression psychotherapy trials (www.metapsy.org) from inception to 1 January 2022. This METAPSY database is developed through comprehensive literature searches in PubMed, PsycINFO, Embase.com, and the Cochrane Library and is updated annually. In each review phase (screening, eligibility, and inclusion), records were screened by two raters independently. Disagreements were resolved through consensus.

We included studies if they reported outcomes on standardized measures of depressed adult participants receiving STPP. Participants were considered depressed if they met specified criteria for major depressive disorder or another unipolar mood disorder as assessed by means of a semi-structured interview or clinicians' assessment, or if they presented an elevated score above the ‘no depression’ cut-off on a standardized measure of depression. We included studies in which STPP (a) was based on psychoanalytic theories and practices, (b) was time-limited from the onset (i.e. not a therapy that was brief only in retrospect), and (c) applied verbal techniques (e.g. therapies applying art as expression form were not considered STPP). Inclusion criteria were assessed at the study level. From the resulting set of studies, we identified randomized comparisons of combined treatment (antidepressants + STPP, provided individually in an outpatient setting) *v.* antidepressants (with/without BSP to control for non-specific treatment effects).

### Data collection

Authors of the included studies were contacted and invited to contribute their studies' IPD. We decided a priori to request anonymized participant-level datasets, including all variables assessed before treatment start (potential moderators) and all outcome variables assessed during and after treatment. We checked whether the IPD received matched the data reported in the publication and whether outcome and moderator variables had out-of-range, invalid, or inconsistent scores (Driessen et al., 2018). We listed multiple study characteristics (Driessen et al., 2015). We rated Cochrane risk of bias items for selection and detection bias based on information in the publications, and attrition bias based on the IPD (Higgins et al., 2011). If information was not reported in the publications, we requested this from the authors. We did not rate performance bias as it is considered impossible to blind participants and therapists to treatment in psychotherapy research. Selective reporting bias was considered not applicable, as we requested all outcome measures assessed.

### Measures

Depression symptom level at post-treatment constituted the pre-specified primary outcome measure. Follow-up was the additional time point. For each trial, we identified the primary continuous depression outcome and post-treatment/follow-up end points as defined by the authors. Because different depression measures were used, we standardized outcomes by converting depression scores into *z*-scores within end point within each study. We conducted sensitivity analyses using unstandardized 17-item Hamilton Depression Rating Scale (HAMD) scores as outcome.

Potential moderators were pre-specified as all pre-treatment demographic, clinical, and psychological participant characteristics that were assessed in more than one study. Based on definitions provided in data dictionaries and/or publications we decided whether variables could be pooled across studies. If constructs were operationalized differently in individual studies, they were standardized too, by converting scores into *z*-scores (within study) for continuous variables or by recoding variables into similar categories for categorical variables (see online Supplementary Table ST1 for an overview). Standardization occurred before data-analysis started and no changes were made afterward.

### Data analysis

We conducted one-stage IPD meta-analyses using mixed-effects models with a three-level structure (study, participant, repeated measures) and restricted maximum likelihood estimation. Analyses were based on intent-to-treat samples. Follow-up data were excluded from post-treatment analyses, due to the fact that additional help-seeking could not be controlled. We estimated heterogeneity with the *I*^2^ statistic.

For each potential moderator, we estimated a model including time and the moderator, a time-by-treatment interaction, and a time-by-moderator-by-treatment three-way interaction. This approach is recommended by Twisk et al. (2018) because it adequately accounts for baseline depression values and has favorable properties concerning missing data (i.e. participants with a baseline value but missing post-treatment and/or follow-up assessments are still included in the analyses). Time was treated as a categorical variable to facilitate treatment comparison at the two end points and continuous moderator variables were grand-mean centered. To account for the clustering of participants within studies, we estimated a random intercept with respect to study. We also estimated a random intercept with respect to participants, and fixed slopes. Based on a −2-log likelihood change evaluation in a basic model (time and time-by-treatment interaction), we added a random slope for the time-by-treatment interaction at the study level in the sensitivity analyses with unstandardized 17-item HAMD scores.

Using this approach, regression coefficients of the time-by-treatment interactions at post-treatment and follow-up represent the treatment comparisons at these assessment moments (Twisk et al., 2018). For analyses with *z*-scores as outcome measure, these regression coefficients are standardized mean differences, which can be interpreted in the same way as a Cohen's *d* effect size. For the analyses with unstandardized 17-item HAMD scores as outcome measure, regression coefficients are mean differences. For categorical moderator variables, we varied the reference category to obtain treatment comparisons in each moderator category. The three-way interaction's regression coefficient reflects the difference between time-by-treatment interactions for different moderator levels. A Bonferroni correction for multiple testing would yield an *α* level of 0.00125 but would also increase the risk of type-II errors given the low power of three-way interactions. We thus considered a *p* value of <0.05 for the three-way interaction indicating a statistically significant moderator effect but interpreted *p* values >0.00125 with caution. We visually inspected histograms of (standardized) residuals after each analysis. The normality assumption was always met.

Next, we conducted three pre-specified sensitivity analyses. First, we repeated the analyses in studies that administrated the 17-item HAMD, using unstandardized scores as outcome measure. Second, to examine the impact of risk of bias in the primary studies, we added risk of bias items as dichotomous covariates to the mixed-effects models (Higgins, Thompson, & Spiegelhalter, 2009). Third, we conducted analyses including only studies that scored negative on all risk of bias criteria assessed. Finally, we modeled all variables with significant three-way interactions simultaneously.

We then conducted two post-hoc sensitivity analyses. To examine the representativeness of the findings of those eligible for STPP for depression, we examined whether significant moderator effects were still present when excluding studies that only included participants with specific comorbidities. Second, we examined the potential subgroup difference of studies with and without BSP in the comparison condition (Driessen et al., 2020) by examining significant moderator effects at follow-up in both subgroups. Mixed-effects model analyses were performed with MLwiN (version 2.26). Data availability bias was assessed by examining differences in study characteristics and effect sizes between studies that contributed IPD and studies that did not (Driessen et al., 2018). We examined publication bias by assessing funnel plot asymmetry for meta-analyses including ⩾10 studies (Sterne et al., 2011).

We also conducted pre-specified exploratory machine learning analyses with the generalized linear mixed-effects model (GLMM) tree algorithm (Fokkema, Smits, Zeileis, Hothorn, & Kelderman, 2018) and cross-validation techniques (Kim, 2009), which are described in detail in Appendix 1 (online Supplementary material). These analyses offer several advantages over traditional mixed-effects models: they allow for detecting non-linear and higher-order interaction effects in nested data, involve less stringent assumptions about the distribution of the data, and result in decision trees that may be easier to interpret and apply in clinical decision making.

## Results

**Fig. 1.**
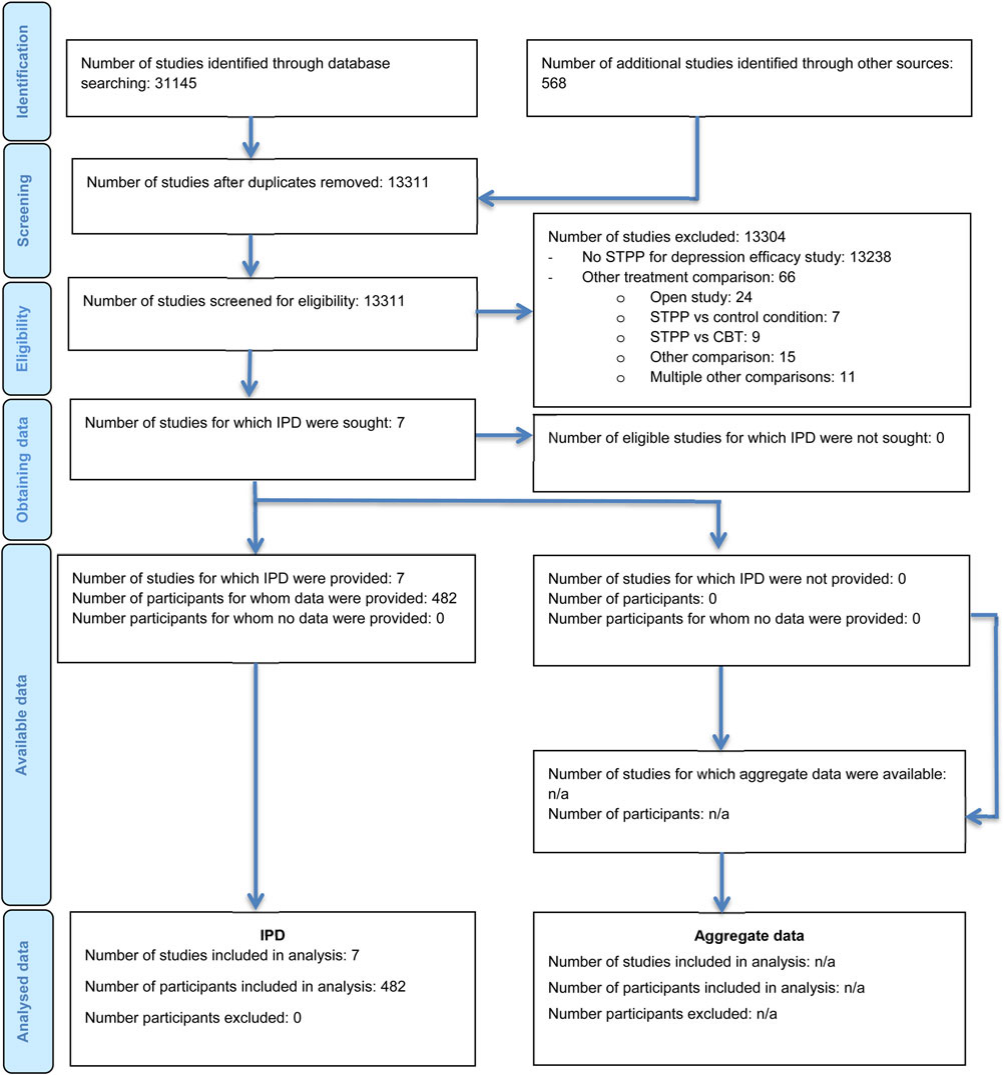
PRISMA-IPD flow diagram. © Reproduced with permission from the PRISMA-IPD Group, which encourages sharing and reuse for non-commercial purposes.

Literature search results are described in Fig. 1. Seven studies were identified to meet the inclusion criteria for this work (Burnand, Andreoli, Kolatte, Venturini, & Rosset, 2002; de Jonghe, Kool, van Aalst, Dekker, & Peen, 2001; López Rodríguez, López Butrón, Vargas Terrez, & Villamil Salcedo, 2004; Maina, Rosso, Crespi, & Bogetto, 2007; Maina, Rosso, Rigardetto, Chiadò Piat, & Bogetto, 2010; Martini, Rosso, Chiodelli, De Cori, & Maina, 2011; Vitriol, Ballesteros, Florenzano, Weil, & Benadorf, 2009). IPD were obtained from all these studies and totaled 482 participants (combined: *n* = 238, 49%; antidepressants: *n* = 244, 51%). Data integrity checks identified inconsistent items for one study and discrepancies between the dataset received and published article for three studies. These were resolved with the authors.

Study characteristics are described in Table 1. Depression inclusion criteria typically consisted of a DSM (Burnand et al., 2002; de Jonghe et al., 2001; López Rodríguez et al., 2004; Maina et al., 2007, 2010) or ICD-10 (Vitriol et al., 2009) depression diagnosis with an elevated HAMD score (Burnand et al., 2002; de Jonghe et al., 2001; Maina et al., 2007, 2010; Vitriol et al., 2009), though in one study (Martini et al., 2011) HAMD score constituted the sole depression inclusion criterion. Four studies (4/7, 57%) included adults with depression in general (Burnand et al., 2002; de Jonghe et al., 2001; López Rodríguez et al., 2004; Maina et al., 2007). Two studies (2/7, 29%) only included participants with specific anxiety disorder comorbidities (Maina et al., 2010; Martini et al., 2011) and one (1/7, 14%) included women with childhood trauma (Vitriol et al., 2009). The antidepressant types and dosages in these three studies did not differ from the standard first-line treatment for depression. STPP was based on various models (Andreoli, 1999; Bellak, 1993, 1994; de Jonghe, Rijnierse, & Janssen, 1994; Malan, 1963, 1976; Safran & Muran, 2000; Vitriol, 2005). Four studies (4/7, 57%) included BSP in the comparison condition (Burnand et al., 2002; Maina et al., 2007; Martini et al., 2011; Vitriol et al., 2009). Six studies (6/7, 86%) used the 17-item HAMD as primary outcome measure (Burnand et al., 2002; De Jonghe et al., 2001; Maina et al., 2007, 2010; Martini et al., 2011; Vitriol et al., 2009). Four studies (4/7, 57%) scored negative on all four risk of bias criteria assessed (de Jonghe et al., 2001; Maina et al., 2007, 2010; Martini et al., 2011; Vitriol et al., 2009).

**Table 1.** Characteristics of the included studies

						Risk of bias assessment			
Study	Antidepressant type^a^	STPP model	STPP weeks	BSP	Follow-up	Seq Gen	All Con	Blind	ITT
Burnand et al. (2002)	1: TCA, 2: SSRI	Safran and Muran (2000), Andreoli (1999)	10	Yes	–	+	+	−	+
de Jonghe et al. (2001)	1: SSRI, 2: TCA, 3: RIMA	de Jonghe et al. (1994)	24	No	1 year	+	+	+	+
López Rodríguez et al. (2004)	SSRI	Bellak (1993, 1994)	26	No	10 months	+	+	+	−
Maina et al. (2007)	SSRI	Malan (1963)	26	Yes	1 year	+	+	+	+
Maina et al. (2010)	SSRI	Malan (1976)	16	No	1 year	+	+	+	+
Martini et al. (2011)	SSRI	Malan (1963)	16	Yes	1 year	+	+	+	+
Vitriol et al. (2009)	1: SSRI/SNRIs, 2: MS, 3: AP	Vitriol (2005)	13	Yes	6 months	+	+	−	+

		Female		Age (years)		Education (years)								Baseline HAMD-17		Episode duration (years)						Comorbid AD	
						0–8		9–12		13–15		>15				<1		1–2		>2			
Study	*n*	*n*	%	Mean	s.d.	*n*	%	*n*	%	*n*	%	*n*	%	Mean	s.d.	*n*	%	*n*	%	*n*	%	*n*	%
Burnand et al. (2002)	81	52	64	36.3	10.1	–	–	–	–	–	–	–	–	24.1	2.9	–	–	–	–	–	–	–	–
de Jonghe et al. (2001)	167	103	62	34.2	9.0	13	8	66	40	71	43	16	10	20.2	4.9	120	72	16	10	25	15	–	–
López Rodríguez et al. (2004)	20	12	60	32.5	9.3	2	10	14	70	0	0	4	20	–	–	–	–	–	–	–	–	–	–
Maina et al. (2007)	35	23	66	35.9	11.1	6	17	10	29	4	11	15	43	20.4	4.6	21	60	13	37	1	3	1	3
Maina et al. (2010)	57	31	54	31.8	7.6	26	46	1	2	22	39	8	14	19.6	5.0	57	100	0	0	0	0	57	100
Martini et al. (2011)	35	29	83	36.1	9.4	5	14	2	6	19	54	9	26	13.1	5.9	–	–	–	–	–	–	35	100
Vitriol et al. (2009)	87	87	100	38.9	11.5	60	69	27	31	0	0	0	0	34.3	6.4	–	–	–	–	–	–	46	53

AD, anxiety disorder; All Con, allocation concealment; AP, second-generation antipsychotic; Blind, blinding of HAMD assessment; BSP, brief supportive psychotherapy in the comparison condition; HAMD-17, 17-item Hamilton Depression Rating Scale; ITT, complete outcome data (full intention-to-treat IPD available); MS, mood stabilizer; RIMA, reversible inhibitor of monoamine-oxidase A; Seq Gen, random sequence generation; SNRI, serotonin noradrenaline reuptake inhibitor; SSRI, selective serotonin reuptake inhibitor; STPP, short-term psychodynamic psychotherapy; TCA, tricyclic antidepressant.

*Note*. –, data not available.

a Numbers represent successive steps in the pharmacotherapy protocol.

Treatment comparisons at post-treatment and follow-up for the different moderator levels are reported in Table 2 (main analyses) and Table ST2 (sensitivity analyses; online Supplementary material). Relevant results for individual studies are reported in Table 3. At post-treatment, baseline HAMD, education, and episode duration were found to moderate treatment effects (Table 2). The effect of adding STPP to antidepressants was larger for participants with high rather than low baseline HAMD scores [*B* = −0.49, 95% confidence interval (CI) −0.61 to −0.37, *p* < 0.0001], for participants with ⩽8 rather than 13–15 education years (*B* = −0.66, 95% CI −1.05 to −0.27, *p* < 0.0009), and for participants with a depressive episode duration of >2 years rather than <1 year (*B* = −0.68, 95% CI −1.31 to −0.05, *p* = 0.03) and than 1–2 years (*B* = −0.86, 95% CI −1.66 to −0.06, *p* = 0.04). No heterogeneity was present in these analyses (*I*^2^ = 0). For baseline HAMD and episode duration, result patterns were replicated across all pre-specified sensitivity analyses (online Supplementary Table ST2). Regarding education, the three-way interaction was no longer statistically significant when considering low risk of bias studies only, nor when modeling all significant moderators simultaneously (*p* = 0.27). The latter model did show significant three-way interactions for baseline HAMD (*p* < 0.001) and episode duration (<1 *v.* >2 years: *p* = 0.003, 1–2 *v.* >2 years: *p* < 0.001). In the post-hoc sensitivity analysis excluding studies that only included participants with specific comorbidities (Maina et al., 2010; Martini et al., 2011; Vitriol et al., 2009), significant moderator effects were also found for baseline HAMD (*p* < 0.0001) and episode duration (<1 *v.* >2 years: *p* = 0.046), although the 1–2 *v.* >2 episode duration year contrast was no longer statistically significant (*p* = 0.0507).

**Table 2.** Cohen's *d* effect sizes of combined treatment of antidepressants and STPP *v.* antidepressants at post-treatment and follow-up for the different moderator levels

	*k*	*n*	Post-treatment			Follow-up		
Moderator levels			*d*	95% CI	*p*	*d*	95% CI	*p*
Gender	7	478						
Male			−0.450	−0.734 to −0.166	0.002	−0.622	−0.918 to −0.326	<0.0001
Female			−0.169	−0.379 to 0.041	0.11	−0.450	−0.670 to −0.230	<0.0001
Marital status	6	397						
Married/cohabiting			−0.295	−0.546 to −0.044	0.02	−0.567	−0.808 to −0.326	<0.0001
Single			−0.311	−0.599 to −0.023	0.03	−0.468	−0.740 to −0.196	0.0008
Divorced/widowed			−0.085	−0.516 to 0.346	0.70	−0.344	−0.767 to 0.079	0.11
Education^a^	6	397						
⩽8 years			**−0.577**	**−0.885 to −0.269**	**0.0002**	**−0.762**	**−1.066 to −0**.**458**	**<0.0001**
9–12 years			−0.221	−0.531 to 0.089	0.16	−0.398	−0.688 to −0.108	0.007
13–15 years			**0.078**	**−0.230 to 0.386**	**0.62**	**−0.225**	**−0**.**517 to 0**.**067**	**0.13**
>15 years			−0.337	−0.790 to 0.116	0.14	**−0.752**	**−1**.**183 to −0**.**321**	**0.0006**
Job status	7	470						
Employed/student			−0.285	−0.508 to −0.062	0.01	−0.522	−0.761 to −0.283	<0.0001
Other			−0.234	−0.489 to 0.021	0.07	−0.516	−0.777 to −0.255	0.0001
Religion	2	187						
No			−0.287	−0.681 to 0.107	0.15	−0.169	−0.532 to 0.194	0.36
Yes			−0.429	−0.958 to 0.100	0.11	−0.617	−1.101 to −0.133	0.01
Episode duration^b^	3	250						
<1 year			**−0.089**	**−0.381 to 0.203**	**0.55**	−0.274	−0.544 to −0.004	0.05
1–2 years			**0.071**	**−0.509 to 0.651**	**0.81**	−0.729	−1.246 to −0.212	0.006
>2 years			**−0.786**	**−1.415 to −0.157**	**0.01**	−0.441	−0.980 to 0.098	0.11
Prior treatment	3	302						
No			−0.311	−0.583 to −0.039	0.03	−0.199	−0.460 to 0.062	0.13
Yes			−0.411	−0.756 to −0.066	0.02	−0.515	−0.842 to −0.188	0.002
Prior depressive episode	5	419						
No			−0.317	−0.584 to −0.050	0.02	−0.486	−0.770 to −0.202	0.0008
Yes			−0.233	−0.480 to 0.014	0.06	−0.415	−0.680 to −0.150	0.002
History of hospitalization	3	302						
No			−0.304	−0.551 to −0.057	0.02	−0.285	−0.522 to −0.048	0.02
Yes			−0.871	−1.496 to −0.246	0.01	−0.603	−1.197 to −0.009	0.05
Comorbid personality disorder	5	363						
No			−0.179	−0.434 to 0.076	0.17	−0.485	−0.757 to −0.213	0.0005
Yes			−0.096	−0.388 to 0.196	0.52	−0.398	−0.706 to −0.090	0.01
Comorbid anxiety disorder^c^	4	211						
No			−0.460	−0.821 to −0.099	0.01	**−0.959**	**−1**.**314 to −0**.**604**	**<0.0001**
Yes			−0.177	−0.457 to 0.103	0.22	**−0.476**	**−0**.**750 to −0**.**202**	**0.0007**
Baseline HAMD	7	479						
Average			−0.204	−0.339 to −0.069	0.003	−0.466	−0.631 to −0.301	<0.0001
Per s.d. increase			**−0.493**	**−0.603 to −0.383**	**<0.0001**	**−0.597**	**−0**.**726 to −0**.**468**	**<0.0001**
Age	7	478						
Average			−0.261	−0.449 to −0.073	0.007	−0.494	−0.694 to −0.294	<0.0001
Per year increase			−0.013	−0.029 to 0.003	0.10	0.002	−0.014 to 0.018	0.80
CGI-S	4	256						
Average			−0.117	−0.354 to 0.120	0.33	−0.426	−0.649 to −0.203	0.0002
Per point increase			0.087	−0.142 to 0.316	0.46	0.093	−0.127 to 0.313	0.41
GAF	3	266						
Average			−0.209	−0.462 to 0.044	0.11	−0.224	−0.530 to 0.082	0.15
Per point increase			0.003	−0.013 to 0.019	0.71	0.007	−0.011 to 0.025	0.44
Comorbid anxiety symptom level	5	376						
Average			−0.267	−0.467 to −0.067	0.009	−0.504	−0.698 to −0.310	<0.0001
Per s.d. increase			−0.026	−0.189 to 0.137	0.75	**−0.233**	**−0**.**390 to −0**.**076**	**0.004**

CGI-S, Clinical Global Impression subscale ‘Severity of Illness’; GAF, Global Assessment of Functioning Scale; HAMD, Hamilton Depression Rating Scale; STPP, short-term psychodynamic psychotherapy.

*Note*. Negative signs indicate lower depressive symptom levels (i.e. better outcomes) in the combined antidepressants and STPP treatment condition than in the comparison condition. Numbers printed in bold indicate statistically significant time-by-moderator-by-treatment three-way interactions (*p* < 0.05). For categorical variables, this indicates a significant difference between the treatment effect in this category and another (see below). For continuous variables, the first effect size (‘Average’) reflects the treatment comparison for participants with baseline scores at the average of the study sample, while the second number (‘Per … increase’) reflects the additional effect for each unit increase in baseline score.

a ⩽8 years *v.* 13–15 years (post-treatment): *B* = −0.66, 95% CI −1.05 to −0.27, *p* < 0.001; ⩽8 years *v.* 13–15 years (follow-up): *B* = −0.54, 95% CI −0.91 to −0.17, *p* = 0.005; 13–15 years *v.* >15 years (follow-up): *B* = −0.53, 95% CI −1.02 to −0.04, *p* = 0.03.

b <1 year *v.* >2 years: *B* = −0.68, 95% CI −1.31 to −0.05, *p* = 0.03; 1–2 years *v.* >2 years: *B* = −0.86, 95% CI −1.66 to −0.06, *p* = 0.04.

c *B* = −0.48, 95% CI −0.87 to −0.09, *p* = 0.01.

**Table 3.** Results of individual studies

	Observed HAMD scores												Moderating effect of baseline HAMD score		
	Post-treatment						Follow-up						Post-treatment		
	Combined			Antidepressants			Combined			Antidepressants					
Study	Mean	s.d.	*n*	Mean	s.d.	*n*	Mean	s.d.	*n*	Mean	s.d.	*n*	*d*	95% CI	*p*
Burnand et al. (2002)	8.94	7.00	35	9.67	7.34	39	–	–	–	–	–	–	−0.544	−0.828 to −0.260	<0.001
de Jonghe et al. (2001)	10.53	6.70	59	13.64	8.44	33	6.78	6.88	60	9.32	9.15	37	−0.644	−0.816 to −0.472	<0.001
López Rodríguez et al. (2004)	0.10	0.32	10	0.30	0.48	10	0.00	0.00	10	0.30	0.48	10	−0.329	−0.982 to 0.324	0.323
Maina et al. (2007)	10.19	3.47	16	9.63	4.08	16	6.06	3.92	16	12.81	4.48	16	0.935	−0.582 to 2.452	0.227
Maina et al. (2010)	13.96	5.69	23	15.96	5.62	27	13.57	5.16	23	15.48	5.54	27	−0.262	−0.550 to 0.026	0.075
Martini et al. (2011)	6.94	4.55	16	7.05	3.79	19	4.50	4.07	16	8.00	4.47	19	0.041	−0.267 to 0.349	0.794
Vitriol et al. (2009)	20.92	8.51	39	26.90	9.47	40	16.56	9.02	36	24.20	10.99	35	−0.566	−0.813 to −0.319	<0.001

	Moderating effect of baseline HAMD score	Treatment effects for different moderator levels
		Follow-up		Episode duration (post-treatment)
		<1 year	1–2 years	>2 years
Study	*d*	95% CI	*p*	*d*	95% CI	*p*	*d*	95% CI	*p*	*d*	95% CI	*p*
Burnand et al. (2002)	–	–	–	–	–	–	–	–	–	–	–	–
de Jonghe et al. (2001)	−0.670	−0.062 to −0.478	<0.001	−0.119	−0.540 to 0.302	0.580	−0.092	−0.992 to 0.808	0.841	−0.865	−1.545 to −0.185	0.013
López Rodríguez et al. (2004)	−0.443	−1.100 to 0.214	0.186	–	–	–	–	–	–	–	–	–
Maina et al. (2007)	0.177	−1.324 to 1.678	0.817	0.011	−0.834 to 0.856	0.980	0.448	−0.524 to 1.420	0.366	–	–	–
Maina et al. (2010)	−0.333	−0.613 to −0.053	0.020	−0.036	−0.497 to 0.425	0.878	–	–	–	–	–	–
Martini et al. (2011)	−0.060	−0.438 to 0.318	0.756	–	–	–	–	–	–	–	–	–
Vitriol et al. (2009)	−0.790	−1.068 to −0.512	<0.001	–	–	–	–	–	–	–	–	–

	Treatment effects for different moderator levels	
	Comorbid anxiety disorder (follow-up)	
	No	Yes	
Study	*d*	95% CI	*p*	*d*	95% CI	*p*	
Burnand et al. (2002)	–	–	–	–	–	–						
de Jonghe et al. (2001)	–	–	–	–	–	–						
López Rodríguez et al. (2004)	–	–	–	–	–	–						
Maina et al. (2007)	−1.416	−2.020 to −0.812	<0.001	−1.731	−3.685 to 0.223	0.083						
Maina et al. (2010)	–	–	–	0.043	−0.353 to 0.439	0.831						
Martini et al. (2011)	–	–	–	−0.681	−1.195 to −0.167	0.009						
Vitriol et al. (2009)	−0.826	−1.389 to −0.263	0.004	−0.540	−1.044 to −0.036	0.036						

–, data not available; HAMD, Hamilton Depression Rating Scale.

*Note*. Negative signs indicate lower depressive symptom levels (i.e. better outcomes) in the combined antidepressants and STPP treatment condition than in the comparison condition.

At follow-up, baseline HAMD, education, anxiety disorder comorbidity, and baseline anxiety symptom level were found to moderate treatment effects. The effect of adding STPP to antidepressants was larger for participants with high rather than low baseline HAMD scores (*B* = −0.60, 95% CI −0.74 to −0.46, *p* < 0.0001), for participants with ⩽8 (*B* = −0.54, 95% CI −0.91 to −0.17, *p* = 0.005) and >15 (*B* = −0.53, 95% CI −1.02 to −0.04, *p* = 0.03) rather than 13–15 education years, for participants without rather than with a comorbid anxiety disorder (*B* = −0.48, 95% CI −0.87 to −0.09, *p* = 0.01), and for participants with high rather than low baseline anxiety symptom levels (*B* = −0.23, 95% CI −0.39 to −0.08, *p* = 0.004). No heterogeneity was present in these analyses (*I*^2^ = 0). For baseline HAMD and anxiety disorder comorbidity, result patterns were replicated across all pre-specified sensitivity analyses. Regarding education, statistically significant three-way interactions were apparent in all pre-specified sensitivity analyses, though the specific contrasts that were significant differed between the analyses. Regarding baseline anxiety symptom level, the three-way interaction was no longer statistically significant when modeling all significant moderators simultaneously (*p* = 0.37). This final model did include significant three-way interactions for baseline HAMD (*p* < 0.001), anxiety disorder comorbidity (*p* = 0.02), and education (⩽8 *v.* 9–12 years: *p* = 0.03; 9–12 *v.* >15 years *p* = 0.03). The significant moderator effect for baseline HAMD was also observed in both post-hoc sensitivity analyses (*p* < 0.0001). The effect for anxiety disorder comorbidity was no longer statistically significant when excluding the three studies that only included participants with specific comorbidities (*p* = 0.75), nor in the subgroup of studies with BSP in the comparison condition (*p* = 0.12, no moderator effect could be estimated in the subgroup of studies without BSP).

GLMM tree analyses' results are presented in Appendix 1 (online Supplementary material) and also identified baseline HAMD, episode duration, and anxiety disorder comorbidity as moderators. Correlations between predicted and observed outcomes for the trees were indicative of small to medium-to-large effects.

## Discussion

We conducted a systematic review and IPD meta-analysis to examine which patients benefit from adding STPP to antidepressants in the treatment of depression. Across the seven studies that were identified by a thorough literature search, we found baseline HAMD and episode duration moderating post-treatment efficacy and baseline HAMD and anxiety disorder comorbidity moderating efficacy at follow-up. Adding STPP to antidepressants was more efficacious for participants with high baseline depression levels, for participants with episode durations of >2 years, and for participants without a comorbid anxiety disorder.

Regarding baseline depression, our findings are in line with work reporting treatment effects to increase with symptom severity for antidepressants (Stone, Kalaria, Richardville, & Miller, 2018), psychotherapy (Driessen, Cuijpers, Hollon, & Dekker, 2010), and the addition of the cognitive behavioral analysis system of psychotherapy to antidepressants (Furukawa et al., 2018). Thus, baseline severity appears to moderate depression treatment efficacy in general rather than applying to STPP specifically. These findings have been taken to imply that relative to low-severity patients, high-severity patients are in more need of treatments with specific effects in order to get well (Driessen et al., 2010).

Concerning episode duration, our findings are in line with studies demonstrating the effects of psychodynamic therapy for patients with treatment resistant depression (Fonagy et al., 2015; Town, Abbass, Stride, & Bernier, 2017) who typically suffer from long-duration episodes. Episode duration has also been observed to moderate the effect of antidepressants combined with STPP *v.* antidepressants combined with cognitive behavioral therapy (CBT; Driessen et al., 2016), such that combined treatment with STPP was more effective for patients with episode durations of ⩾1 year. It has been speculated in this regard that individuals with longer episode durations have depressive symptoms that are more influenced by their personality structure resulting in more complex working alliances and transference feelings; psychodynamic therapists are trained to elaborate on these therapeutic relational aspects if necessary (Driessen et al., 2016). However, the strength of evidence for episode duration as a moderator is limited by the small number of participants reporting chronic episodes.

Concerning anxiety disorder comorbidity, we also found some indication for a moderating effect at post-treatment, but this was only significant in one sensitivity analysis. The strength of evidence for anxiety disorder comorbidity as a moderator at follow-up is limited by the lack of variability in three of the four studies assessing this variable, suggesting that this finding might be driven by between-study effects, and the *p* values exceeding the Bonferroni correction. Finally, education was associated with treatment efficacy in the mixed-effects models. The specific contrasts between the four education levels, however, reached statistical significance in some analyses but not in others, and education did not appear as a moderator in the GLMM trees.

### Strengths and limitations

This study has a number of strengths. First, to the best of our knowledge, this is the first study that examines moderators across randomized clinical trials that assess the efficacy of adding STPP to antidepressants. Second, this meta-analysis did not suffer from data availability bias as IPD were obtained for all included studies. Risk of selection bias in the primary studies was also low. Third, the included studies shared similarities in depression inclusion criteria and primary outcome measure. Fourth, we used mixed-effects models as well as a novel tree-based machine learning technique to examine moderators at both post-treatment and follow-up. This allowed for identifying both short- and long-term moderator effects, as well as potential higher-order interactions. The GLMM trees (Figs. SA1 and SA2, Appendix 1, online Supplementary material) may be easier to apply in clinical practice. Fifth, using IPD meta-analytic methods increased the statistical power to examine moderators (Lambert et al., 2002).

This study also has a number of limitations. First, even though IPD could be obtained for all studies, the total number of participants included in this meta-analysis is modest. Relatively few studies examined the efficacy of adding STPP to antidepressants and all were conducted more than 10 years ago. We think this reflects that psychodynamic therapy in general has been studied less extensively than other forms of psychotherapy for depression, such as CBT (e.g. Cuijpers et al., 2021). Although this study was adequately powered to identify certain moderators, it might have lacked statistical power to identify relatively small moderator relationships or higher-order interactions. Related, not all moderator variables were assessed in all studies. Thus, the individual moderator models can relate to different subgroups of studies. Second, not all studies were free from detection and attrition bias, though the moderator findings discussed previously appeared robust against controls for these risks of bias. The number of studies included in this meta-analysis was too small to assess publication bias. Third, although the studies shared similarities, they also differed with regard to the STPP model used, the antidepressant type, and follow-up length, for instance. Regardless of these differences, moderator effects could be identified in the combined studies' data. Fourth, we were not able to examine every potential moderator variable of interest (e.g. personality structure), because they were not assessed in the primary studies. Fifth, and most important, these findings are observational and need validation in independent samples. Although we applied cross validation to estimate predictive accuracy for the GLMM trees, further replication of our findings would strengthen the basis for their use in guiding treatment selection.

### Clinical and research implications

The findings of this study suggest that adding STPP to antidepressants might be particularly efficacious for individuals with relatively high baseline HAMD scores, for individuals with episode durations of >2 years (at post-treatment), and for those without a comorbid anxiety disorder (at follow-up). For individuals with relatively low baseline HAMD scores, for individuals with episode durations of ⩽2 years, and for individuals with a comorbid anxiety disorder, adding STPP might not result in superior treatment effects. However, the findings of this study cannot be taken to imply that antidepressants only (or combined with BSP) should be considered the first-choice treatment for these latter individuals, as this study does not speak to the comparative efficacy of antidepressants *v.* other depression treatments (e.g. CBT). In addition, this study does not speak to which patients benefit from adding antidepressants to STPP, a question that is as relevant, but is not possible to address with an IPD meta-analysis yet due to a lack of randomized controlled trials.

Given the clinical importance of the research question and the limitations of the current study in terms of sample size and moderator variables assessed, further study of which patients benefit from adding STPP to antidepressants in the treatment of depression is warranted. Specifically, the field would benefit from additional large-scale rigorously conducted randomized clinical trials that include BSP in the comparison condition. Comparisons of combined treatment with STPP only are also needed to address the (reversed) question of which patients benefit from adding antidepressants to STPP. Future trials should assess a range of potential moderators, including baseline depression severity, episode duration, and anxiety disorder comorbidity, but also education level and personality structure. Data from such future trials would provide an important validation set for the current findings. If validated, the findings of this study would suggest that depression severity, episode duration, and anxiety disorder comorbidity are important factors to consider when adding STPP to antidepressants. Such knowledge can be used to facilitate evidence-based personalized treatment selection for depression and making more efficient use of existing depression treatment resources.

## Data Availability

The collective de-identified individual participant database developed for this study, as well as a data dictionary and relevant related documents (e.g. study protocol) are available for use by other researchers with publication of this manuscript. Requests can be made with the corresponding author (ellen.driessen@ru.nl). Access (with limited investigator support) will be granted after approval of a study proposal by all authors and a signed data access agreement.

## Supplementary material

For supplementary material accompanying this paper visit https://doi.org/10.1017/S0033291722003270. click here to view supplementary material
