# Supplementary material for: Which patients benefit from adding short-term psychodynamic psychotherapy to antidepressants in the treatment of depression? A systematic review and meta-analysis of individual participant data
Source: Psychol Med. 2022 Nov 21;53(13):6090–101. doi: 10.1017/S0033291722003270 (PMC10520584; doi:10.1017/S0033291722003270)
Supplement: Supplementary file 1 [file S0033291722003270sup001.docx]

**Supplementary material**

**Appendix 1**

**Exploratory generalized linear mixed-effects model (GLMM) tree analyses**

GLMM tree analyses were performed in R (version 3.6.1; R Core Team, 2019) using package glmertree (version 0.2-0; Fokkema, Smits, Zeileis, Hothorn, & Kelderman, 2018). We specified GLMM trees so that a global random intercept was estimated with respect to participants. Furthermore, local (i.e., subgroup-specific) models were estimated including time and the time-by-treatment interaction. Potential moderator variables were used as partitioning variables to identify subgroups for which the estimated time-by-treatment interaction effects differed. Age, gender, and baseline Hamilton Depression Rating Scale (HAMD) were included as possible partitioning variables in all analyses, while the other variables were added separately to minimize listwise deletion of observations with missing values. To correct for the effect of multiple testing, a Bonferroni correction was applied to the p-values when determining the statistical significance of the potential moderator variables’ effects. We tested statistical significance of treatment effect comparisons in the tree nodes using package lmerTest (version 3.1-0; Kuznetsova, Brockhoff, & Christensen, 2017). As this does not account for the searching of the tree structure, and thus may result in underestimated standard errors and p-values, we employed a more conservative criterion and interpreted p-values <0.01 to indicate significant treatment effects.

**Results of the GLMM tree analyses**

GLMM trees are represented in Figures SA1 and SA2, for analyses with HAMD z-scores and with unstandardized 17-item HAMD scores as outcome measures, respectively.

Analyses with HAMD z-scores as outcome measure identified episode duration as a moderator of treatment effect (Figure SA1), such that adding STPP to antidepressants was more efficacious at post-treatment for participants with a depressive episode duration of >2 years rather than ≤2 years.

Analyses with unstandardized 17-item HAMD scores as outcome measure identified baseline HAMD and anxiety disorder comorbidity as moderators of treatment effect (Figure SA2). In the analysis with baseline HAMD, age, and gender as potential moderators (Figure SA2, panel A), the benefit of adding STPP increased with baseline depression severity. This effect was statistically significant and large at post-treatment and follow-up for participants with baseline HAMD scores of >36, statistically significant but smaller for participants scoring 17-36, and not statistically significant for participants with baseline HAMD scores of ≤16. When adding anxiety disorder comorbidity, the tree suggested a higher-order interaction between this variable and baseline HAMD (Figure SA2, panel B). In the group of participants with baseline HAMD scores of ≤35, no significant effect of adding STPP to antidepressants was found at follow-up for participants with a comorbid anxiety disorder, while a significant effect was apparent for participants without such a comorbidity.

The effect of episode duration on HAMD z-scores (Figure SA1) was not fully replicated on the unstandardized 17-item HAMD scores. While episode duration showed the strongest evidence, compared to HAMD baseline scores, age, and gender, the parameter stability test did not reach statistical significance after Bonferroni correction (*p*=0.04). The effect of baseline HAMD on unstandardized 17-item HAMD scores (Figure SA2A) was not fully replicated on the HAMD z-scores. While HAMD baseline scores showed the strongest evidence for being a moderator variable, the parameter stability test did not reach statistical significance after Bonferroni correction (*p*=0.01). The effect of baseline HAMD and comorbid anxiety disorder on unstandardized 17-item HAMD scores (Figure SA2B) was also not fully replicated on the HAMD z-scores. While HAMD baseline scores showed the strongest evidence, followed by anxiety disorder comorbidity, for being a moderator variable in these analyses, the parameter stability tests again did not reach statistical significance after Bonferroni correction (*p*=0.03 and 0.04, respectively). Thus, although the effect of episode duration was not replicated on unstandardized 17-item HAMD scores, and the effects of baseline HAMD and comorbid anxiety disorder were not replicated on HAMD z-scores, they showed the strongest evidence of effect in those analyses but did not reach statistical significance (after Bonferroni correction).

The GLMM tree analyses indicated that the main predictor of treatment outcome differences is baseline HAMD scores, complemented by anxiety disorder comorbidity and episode duration. These variables were also the strongest moderators found in the mixed-effects analyses. In the traditional mixed-effects models, the moderating effect of the variables was tested one-by-one. In the GLMM tree analyses, age, gender, and baseline HAMD were included as possible partitioning variables in all analyses, while the other variables were added separately. This allowed baseline HAMD being picked up instead of other moderators, when this was a stronger predictor of treatment outcome differences. Furthermore, the GLMM tree analyses allow for identifying unspecified higher-order interactions, while the mixed model analyses do not. This explains why the GLMM tree analyses suggested the moderating effect of comorbid anxiety disorder to be present only in the group of participants with HAMD scores of 35 or less.

**Figure SA1. Generalized linear mixed-effects model tree with HAMD z-scores as outcome measure.**


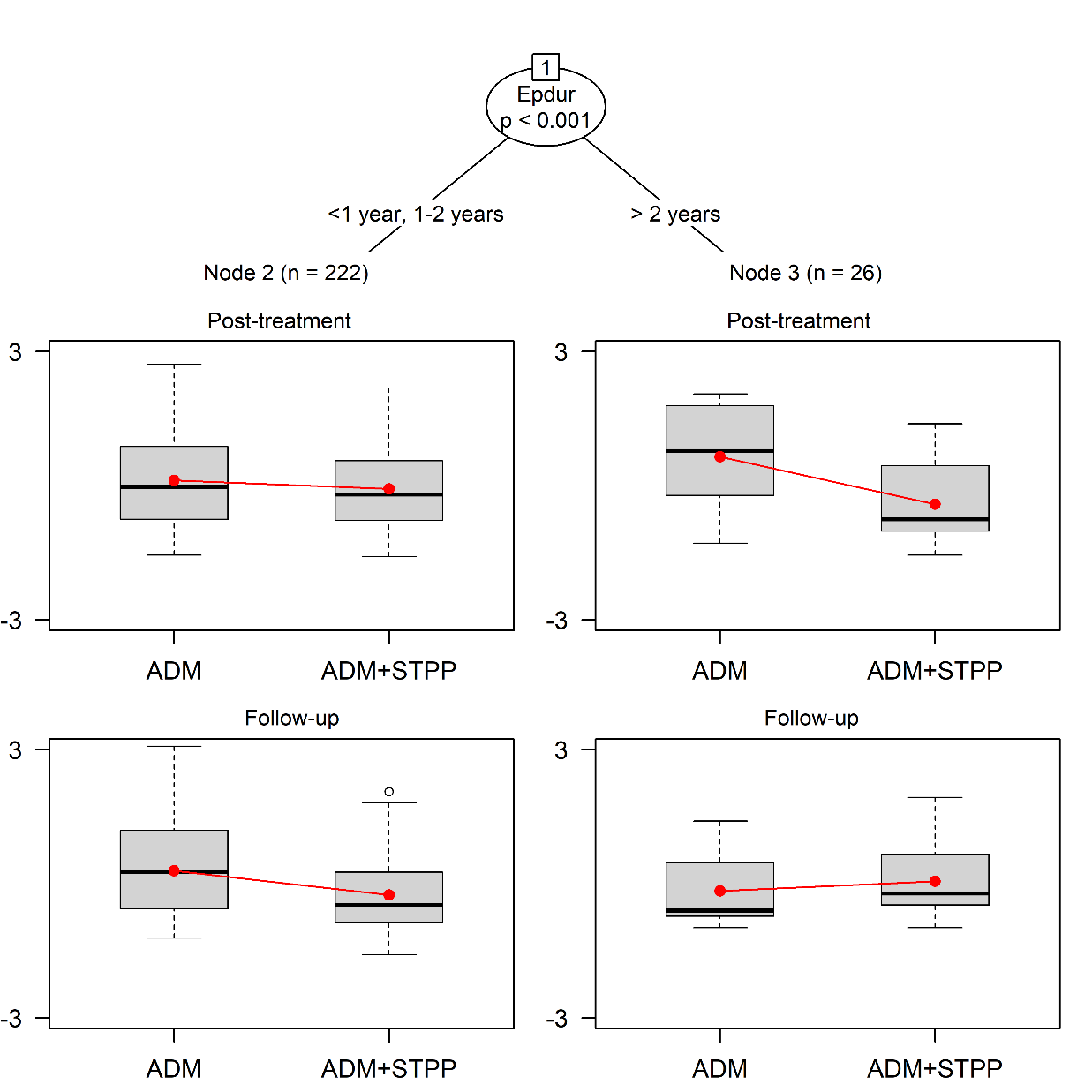


|  | ***d*** | **SE** | ***p*** |  | ***d*** | **SE** | ***p*** |
| --- | --- | --- | --- | --- | --- | --- | --- |
| **Post-treatment** | -0.01 | 0.14 | 0.93 |  | -1.12 | 0.43 | 0.01 |
| **Follow-up** | -0.37 | 0.14 | 0.01 |  | -0.23 | 0.39 | 0.56 |

Note. ADM = antidepressants with/without brief supportive psychotherapy; ADM+STPP = combined treatment of antidepressants and short-term psychodynamic psychotherapy; Epdur = depressive episode duration. *n*s represent the number of participants in each terminal node for whom post-treatment and/or follow-up assessments were available. The y-axes of the boxplots represent Hamilton Depression Rating Scale z-scores. Boxplots represent observed scores for both groups; the dots connected by a line represent the model-predicted averages in the two groups. The tables below terminal nodes represent treatment effects, with *d* = Cohen’s *d* effect size; SE = standard error; *p* = p-value. Negative signs indicate lower depressive symptom levels (i.e., better outcomes) in the combined antidepressants and STPP treatment condition than in the comparison condition. In node 3 at follow-up, the red line has a different direction than the effect size in the table, because the former is computed by marginalizing over the random effects, while the latter is computed by conditioning on the random effects.

**Figure SA2. Generalized linear mixed-effects model trees with unstandardized 17-item HAMD scores as outcome measure.**

1. *Tree for gender, age, and baseline HAMD as possible partitioning variables*


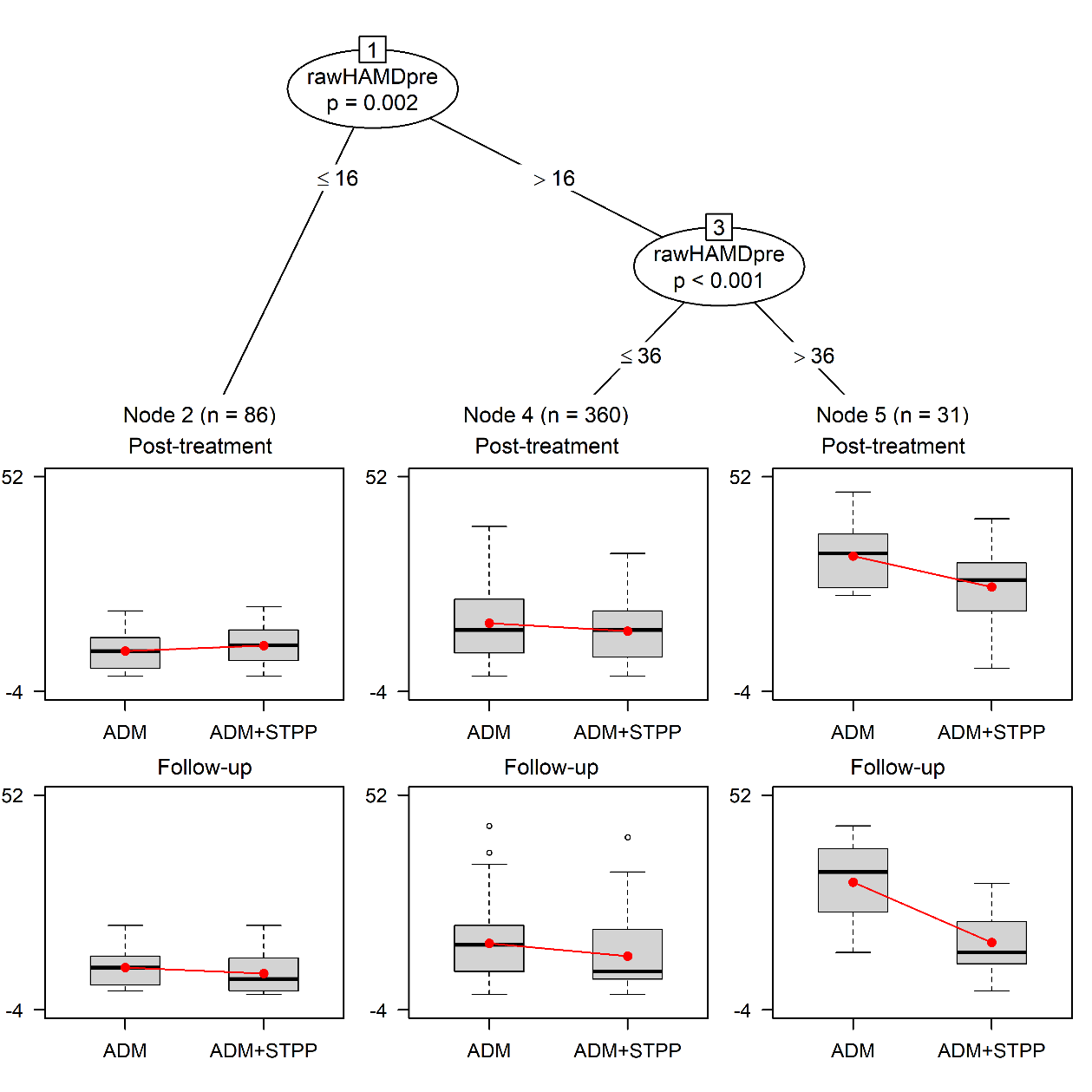


|  | **B** | **SE** | ***p*** |  | **B** | **SE** | ***p*** |  | **B** | **SE** | ***p*** |
| --- | --- | --- | --- | --- | --- | --- | --- | --- | --- | --- | --- |
| **Post-treatment** | 0.54 | 1.51 | 0.72 |  | -1.87 | 0.66 | 0.005 |  | -7.95 | 2.10 | <0.001 |
| **Follow-up** | -2.34 | 1.49 | 0.12 |  | -3.09 | 0.76 | <0.001 |  | -15.07 | 2.26 | <0.001 |

1. *Tree for gender, age, baseline HAMD, and anxiety disorder comorbidity as possible partitioning variables*


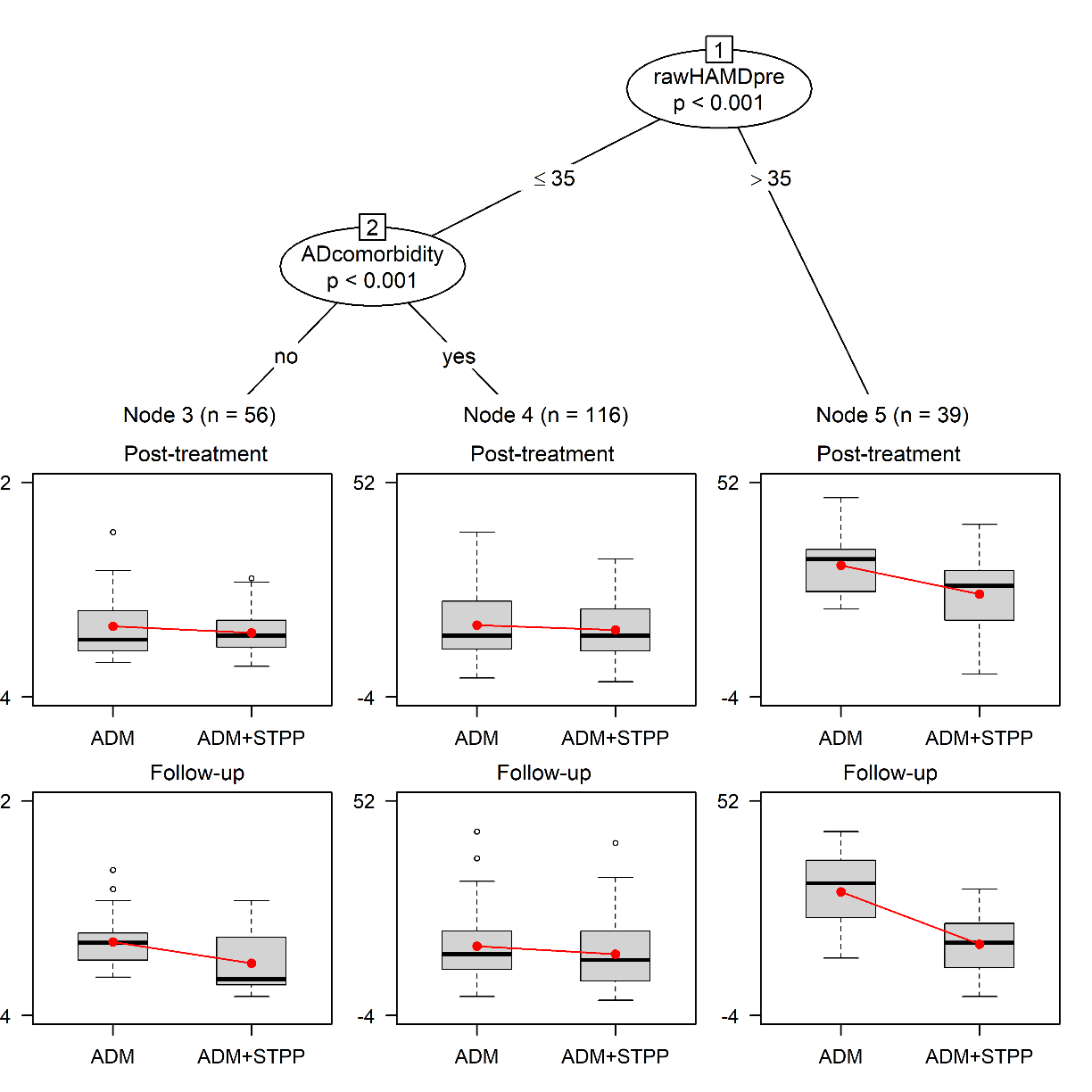


|  | **B** | **SE** | ***p*** |  | **B** | **SE** | ***p*** |  | **B** | **SE** | ***P*** |
| --- | --- | --- | --- | --- | --- | --- | --- | --- | --- | --- | --- |
| **Post-treatment** | -2.87 | 1.62 | 0.08 |  | -0.83 | 1.08 | 0.44 |  | -7.62 | 1.90 | <0.001 |
| **Follow-up** | -6.43 | 1.64 | <0.001 |  | -1.88 | 1.09 | 0.08 |  | -12.99 | 2.03 | <0.001 |

Note. AD = anxiety disorder; ADM = antidepressants with/without brief supportive psychotherapy; ADM+STPP = combined treatment of antidepressants and short-term psychodynamic psychotherapy; HAMD = Hamilton Depression Rating Scale; rawHAMDpre = unstandardized 17-item Hamilton Depression Rating Scale baseline score. *n*s represent the number of participants in each terminal node for whom post-treatment and/or follow-up assessments were available. The y-axes of the boxplots represent unstandardized 17-item Hamilton Depression Rating Scale scores. Boxplots represent observed scores for both groups; the red dots connected by a line represent the model-predicted averages in the two groups. The tables below terminal nodes represent treatment effects, with B = mean difference; SE = standard error; *p* = p-value; . Negative signs indicate lower depressive symptom levels (i.e., better outcomes) in the combined antidepressants and STPP treatment condition than in the comparison condition.

**Stability of the selected splits in the GLMM trees**

We assessed the stability of the fitted GLMM trees using the stablelearner package (version 0.1-1; Philipp, Zeileis, & Strobl, 2016). That is, we fitted GLMM trees on 500 random subsamples comprising 80% of the original data and counted the frequency with which each of the possible partitioning variables was selected for splitting. The results are presented in Table SA1.

For the subsamples drawn from the data used to grow the GLMM tree in Figure SA1 (which has HAMD z-scores as the outcome), episode duration was the only partitioning variable selected in the full sample, and it was selected in the majority of subsamples (277/500, 55%). In some subsamples, multiple splits were made using episode duration, yielding a slightly higher average number of times the variable was selected per subsample (293/500, 59%). Table SA1 further indicates that gender and age were rarely selected, and baseline HAMD score never.

For the GLMM trees in Figure SA2 (which have unstandardized 17-item HAMD scores as the outcome), unstandardized baseline HAMD scores are selected for splitting in the majority of subsamples (> 89%, 445/500) and are often used for multiple splits in the same tree, yielding an average number of times selected per subsample of >1. Age and gender were rarely selected. When anxiety disorder comorbidity was added as a possible partitioning variable (Figure SA2B), it was selected in 52% (261/500) of the subsamples, and age and gender were very rarely selected.

The distribution of splitting values selected for the unstandardized 17-item baseline HAMD scores in Figure SA2 (panels A and B) are presented in Figures SA3 and SA4, respectively. Both histograms indicate a bimodal distribution, with the two modes corresponding to the splitting values appearing in the GLMM trees fitted on the full dataset (FigureSA2, panel A).

| **Table SA1. Selected splitting variables in 500 random subsamples of the original dataset.** | | | |
| --- | --- | --- | --- |
|  |  | **Proportion of subsamples in which variable is selected for splitting** | **Average number of times selected per subsample** |
| **Figure SA1** | Episode duration | 0.554 | 0.586 |
|  | Gender | 0.058 | 0.058 |
|  | Age | 0.010 | 0.014 |
|  | Baseline HAMD | 0.000 | 0.000 |
| **Figure SA2A** | Baseline HAMD | 0.952 | 1.696 |
|  | Age | 0.090 | 0.096 |
|  | Gender | 0.018 | 0.018 |
| **Figure SA2B** | Baseline HAMD | 0.890 | 1.214 |
|  | AD comorbidity | 0.522 | 0.522 |
|  | Age | 0.012 | 0.012 |
|  | Gender | 0.002 | 0.002 |

Note. AD = anxiety disorder; HAMD = Hamilton Depression Rating Scale

| 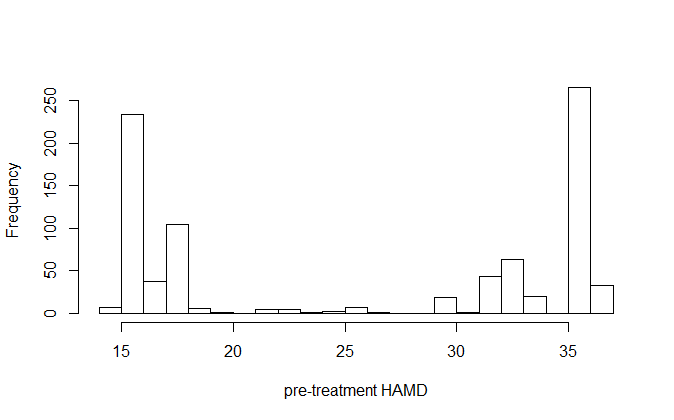**Figure SA3. Distribution of splitting values for the unstandardized 17-item HAMD baseline score for the stability analyses of Figure SA2A.** |
| --- |
| Note. HAMD = Hamilton Depression Rating Scale |
| **Figure SA4. Distribution of splitting values for the unstandardized 17-item HAMD baseline score for the stability analyses of Figure SA2B.** |
| 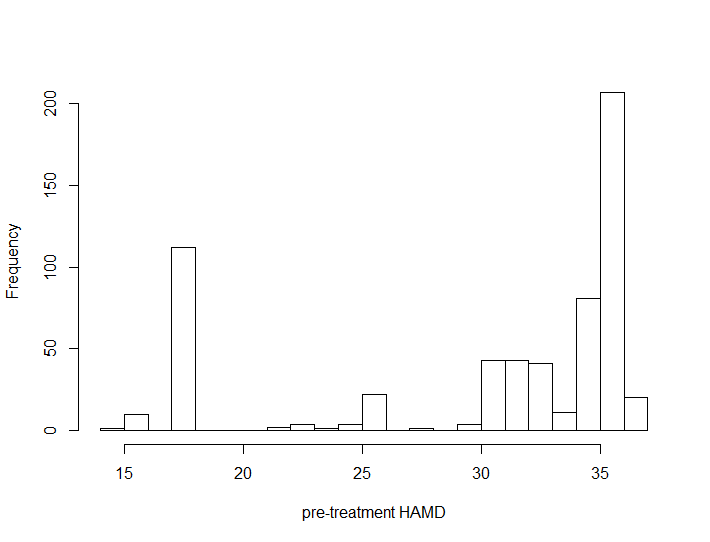 |
| Note. HAMD = Hamilton Depression Rating Scale |

**Predictive accuracy of the GLMM trees**

We estimated expected predictive accuracy on new observations using repeated 10*-*fold cross validation (Kim, 2009). That is, we randomly assigned participants in the original dataset to 1 of 10 approximately equally sized folds. Each of these folds served as a test dataset, for which predictions were computed using a model build on the observations in the remaining 9 folds. This procedure yields a cross-validated prediction of the response variables (pre-treatment, post-treatment, and follow-up HAMD scores) for each participant in the original dataset. Predictions were based on the tree only; that is, random effects were excluded from the predictions, and pre-treatment HAMD levels were only included in the predictive model if they were selected for splitting in the tree.

We computed the correlation between predicted and observed HAMD scores at both post-treatment and follow-up. To reduce the dependence of the results on a single partition of the data into folds, we repeated the 10-fold cross validation 10 times. This approach is known as repeated cross validation and has been found to provide more reliable estimates of predictive performance (Kim, 2009). This yields ten correlation coefficients, over which we calculated the mean and standard deviation, the latter can be interpreted as a standard error of the mean correlation.

The estimated correlations are presented in Table SA2. The results indicate that predictions for unstandardized HAMD scores (Figure SA2) are more accurate than predictions for standardized HAMD scores (Figure SA1). The predictive accuracy of the GLMM trees in Figure SA2, which were fitted using to unstandardized HAMD scores, are indicative of a medium to large effect. The GLMM tree based on standardized HAMD scores (Figure SA1) showed weaker correlations, indicative of a small effect.

| **Table SA2. Cross-validated predictive accuracies for the GLMM trees.** | | |
| --- | --- | --- |
| **GLMM tree** | **Post-treatment HAMD** | **Follow-up HAMD** |
| Figure SA1 | 0.077  (0.061) | 0.145  (0.051) |
| Figure SA2A | 0.465  (0.022) | 0.405  (0.028) |
| Figure SA2B | 0.452  (0.061) | 0.334  (0.034) |
| Note. GLMM = generalized linear mixed-effects model; HAMD = Hamilton Depression Rating Scale. Values represent average correlations between observed and predicted HAMD scores, computed over 10 repeats of 10-fold cross validation. Values within parentheses represent standard errors, i.e., the standard deviation over the 10 repeats. | | |

**References cited:**

R Core Team (2019). R: A language and environment for statistical computing. R Foundation for Statistical Computing, Vienna, Austria. https://www.R-project.org/

Fokkema, M., Smits, N., Zeileis, A., Hothorn, T., & Kelderman, H. (2018). Detecting treatment-subgroup interactions in clustered data with generalized linear mixed-effects model trees. *Behavior Research Methods*, *50*(5), 2016–2034. https://doi.org/10.3758/S13428-017-0971-X

Kim, J. H. (2009). Estimating classification error rate: Repeated cross-validation, repeated hold-out and bootstrap. *Computational Statistics & Data Analysis*, *53*(11), 3725–3745. https://doi.org/10.1016/j.csda.2009.04.009

Kuznetsova, A., Brockhoff, P. B., Christensen, R. H. B. (2017). lmerTest package: Tests in linear mixed effects models. Journal of Statistical Software, *82*(13), 1–26. https://doi.org/ 10.18637/jss.v082.i13

Philipp, M., Zeileis, A., & Strobl, C. (2016). *A toolkit for stability assessment of tree-based learners* (No. 2016-11). Working Papers in Economics and Statistics.

**Table ST1. Overview of moderator variables assessed in the included studies**

| **Variable** | **Burnand, 2002** | **de Jonghe, 2001** | **Lopez Rodriguez, 2004** | **Maina, 2007** | **Maina, 2010** | **Martini, 2011** | **Vitriol, 2009** | **Final coding** |
| --- | --- | --- | --- | --- | --- | --- | --- | --- |
| Gender | Male (0)  Female (1) | Male (0)  Female (1) | Male (0)  Female (1) | Male (0)  Female (1) | Male (0)  Female (1) | Male (0)  Female (1) | Female (1) | 0=Male  1=Female |
| Age | in years | in years | in years | in years | in years | in years | in years | in years |
| Marital Status | - | Single (1)  Divorced (2)  Widowed (2)  LAT (0)  Cohabiting (0)  Married (0) | Married (0)  Single (1)  Divorced/separated (2) | Married (0)  Single (1)  Widowed (2)  Divorced (2) | Married (0)  Single (1)  Divorced/Widow (2) | Married (0)  Single (1)  Widowed (2)  Divorced (2) | Married (0)  Cohabiting (0)  Divorced (2)  Single (1)  Widow (2) | 0=Married/Cohabiting  1=Single  2=Divorced/Separated  /Widowed |
| Education | - | School level + graduated yes/no | Number of years | Number of years | 0-8 years (0)  9-11 years (1)  12 years (1)  13-15 years (2)  >15 years (3) | Number of years | School level + course year | 0= 0-8 years  1= 9-12 years  2= 13-15 years  3= >15 years |
| Job Status | Employed or student (0)  Other (1) | Full-time job (0)  Part-time job (0)  Illness benefit (1)  Benefits (1)  Retirement (1)  Elderly benefits (1)  Student (0) | Labor with financial remuneration:  Yes (0)  No (1) | Full-time employed (0)  Unemployed (1)  Retired (1)  Student (0)  Housewife (1) | Employed/Student (0)  Unemployed/benefits (1) | Full-time employed (0)  Unemployed (1)  Retired (1)  Student (0)  Housewife (1) | Employed (0)  Unemployed (1)  Temporary employed (0)  Student (0)  Housewife (1) | 0=Employed/Student  1=Other |
| Religion | - | Catholic (1)  (Dutch)Reformed (1)  Christian (1)  Islam (1)  Hindu/Buddhist (1)  Jewish (1)  Other (1)  None (0) | Yes (1)  No (0) | - | - | - | - | 0=No  1=Yes |
| Episode duration | - | < 4 weeks (0)  4 weeks–1 year (0)  1-2 years (1)  >2 years (2) | - | <1 years (0)  1-2 years (1)  >2 years (2) | Number of weeks | - | - | 0= <1 year  1= 1-2 years  2= >2 years |
| Prior treatment | - | Psychiatric or outpatient treatment:  Yes (1)  No (0) | - | - | Psychotherapy:  Yes (1)  No (0) | - | Any treatment:  Yes (1)  No (0) | 0=No  1=Yes |
| Prior episode | Past major depressive episode:  Presence (1)  Absence (0) | Number of depressive episodes in the last 5 years | - | Single episode (0)  Recurrent episode (1) | Prior diagnosis:  Yes (1)  No (0) | - | - | 0=No  1=Yes |
| History of hospitalization | - | Number of hospitalizations | - | - | Yes (1)  No (0) | - | Yes (1)  No (0) | 0=No  1=Yes |
| Personality disorder comorbidity | DSM-III-R diagnosis (IPDE):  Yes (1)  No (0) | Screening diagnosis:  Yes (1)  No (0) | - | Diagnosis:  Yes (1)  No (0) | Diagnosis:  Yes (1)  No (0) | Diagnosis:  Yes (1)  No (0) | - | 0=No  1=Yes |
| Anxiety disorder comorbidity | - | - | - | Comorbid panic disorder:  Yes (1)  No (0) | - | Comorbid panic disorder:  Yes (1)  No (0) | Comorbid PTSD (CIDI):  Yes (1)  No (0) | 0=No  1=Yes |
| CGI-S | - | CGI-S score | - | CGI-S score | CGI-S score | CGI-S score | - | CGI-S score |
| GAF | GAF score | GAF score | - | - | GAF score | - | - | GAF score |
| Anxiety symptom level | - | - | - | Hamilton Rating Scale for Anxiety total score | - | Hamilton Rating Scale for Anxiety total score | Post-traumatic Stress Treatment Outcome scale (PTO8) total score | Z-score within study |

Note. CGI-S = Clinical Global Impression subscale ‘Severity of Illness’; CIDI = Composite International Diagnostic Interview; GAF = Global Assessment of Functioning Scale; IPDE = International Personality Disorder Examination; LAT = living apart together; PTSD = post-traumatic stress disorder. Values in parentheses represent the category into which this level was recoded.

**Table ST2. Effects of combined treatment of antidepressants and STPP versus antidepressants at post-treatment and follow-up at the different moderator levels in the three pre-specified sensitivity analyses**.

|  | **1. Unstandardized 17-item HAMD as outcome*** | | | | | | **2. Risk of bias items added as covariates** | | | | | | **3. Low risk of bias studies included only** | | | | | |
| --- | --- | --- | --- | --- | --- | --- | --- | --- | --- | --- | --- | --- | --- | --- | --- | --- | --- | --- |
|  | *Post-treatment* | | | *Follow-up* | | | *Post-treatment* | | | *Follow-up* | | | *Post-treatment* | | | *Follow-up* | | |
|  | B | SE | *p* | B | SE | *p* | *d* | SE | *p* | *d* | SE | *p* | *d* | SE | *p* | *d* | SE | *p* |
| Gender |  |  |  |  |  |  |  |  |  |  |  |  |  |  |  |  |  |  |
| - Male | -2.833 | 1.672 | .09 | -4.443 | 1.766 | .01 | -0.451 | 0.146 | .002 | -0.623 | 0.151 | <.001 | **-0.326** | **0.164** | **.05** | -0.544 | 0.153 | <.001 |
| - Female | -1.075 | 1.521 | .48 | -3.331 | 1.571 | .03 | -0.169 | 0.107 | .11 | -0.450 | 0.112 | <.001 | **0.034** | **0.139** | **.81** | -0.345 | 0.131 | .008 |
| Marital status |  |  |  |  |  |  |  |  |  |  |  |  |  |  |  |  |  |  |
| - Married/cohabiting | -2.129 | 1.715 | .21 | -4.892 | 1.632 | .003 | -0.296 | 0.128 | .02 | -0.569 | 0.123 | <.001 | -0.097 | 0.154 | .53 | -0.497 | 0.144 | <.001 |
| - Single | **-2.711** | **1.765** | **.12** | -2.756 | 1.686 | .10 | -0.315 | 0.147 | .03 | -0.471 | 0.139 | <.001 | -0.176 | 0.154 | .25 | -0.402 | 0.144 | .005 |
| - Divorced/widowed | **0.625** | **2.077** | **.76** | -2.427 | 2.089 | .25 | -0.081 | 0.220 | .71 | -0.338 | 0.216 | .12 | 0.187 | 0.288 | .52 | -0.140 | 0.281 | .62 |
| Education |  |  |  |  |  |  |  |  |  |  |  |  |  |  |  |  |  |  |
| - ≤ 8 years | **-5.255** | **1.025** | **<.001** | **-6.469** | **1.909** | **<.001** | **-0.571** | **0.156** | **<.001** | **-0.757** | **0.154** | **<.001** | -0.317 | 0.229 | .17 | -0.606 | 0.222 | .006 |
| - 9-12 years | **-3.213** | **1.106** | **.004** | **-2.536** | **1.882** | **.18** | -0.222 | 0.158 | .16 | -0.399 | 0.148 | .007 | -0.224 | 0.193 | .25 | -0.454 | 0.177 | .01 |
| - 13-15 years | **0.300** | **1.024** | **.77** | **-1.727** | **1.844** | **.35** | **0.077** | **0.157** | **.62** | **-0.225** | **0.149** | **.13** | 0.155 | 0.157 | .32 | **-0.185** | **0.148** | **.21** |
| - >15 years | **-1.512** | **1.541** | **.33** | -3.680 | 2.169 | .09 | -0.340 | 0.231 | .14 | **-0.753** | **0.220** | **<.001** | -0.269 | 0.231 | .24 | **-0.747** | **0.218** | **<.001** |
| Job status |  |  |  |  |  |  |  |  |  |  |  |  |  |  |  |  |  |  |
| - Employed/student | -1.998 | 1.482 | .18 | -3.829 | 0.881 | <.001 | -0.286 | 0.114 | .01 | -0.523 | 0.122 | <.001 | -0.143 | 0.144 | .32 | -0.456 | 0.135 | <.001 |
| - Other | -1.049 | 1.569 | .50 | -5.031 | 0.981 | <.001 | -0.235 | 0.130 | .07 | -0.517 | 0.133 | <.001 | -0.117 | 0.164 | .48 | -0.471 | 0.152 | .002 |
| Religion |  |  |  |  |  |  |  |  |  |  |  |  |  |  |  |  |  |  |
| - No | -2.654 | 1.256 | .03 | -1.675 | 1.262 | .18 | -0.294 | 0.201 | .14 | -0.173 | 0.185 | .35 | -0.251 | 0.205 | .22 | -0.118 | 0.188 | .53 |
| - Yes | -1.933 | 2.183 | .38 | -3.698 | 2.140 | .08 | -0.426 | 0.270 | .11 | -0.615 | 0.247 | .01 | -0.433 | 0.355 | .22 | -0.660 | 0.318 | .04 |
| Episode duration |  |  |  |  |  |  |  |  |  |  |  |  |  |  |  |  |  |  |
| - <1 year | **-1.440** | **1.802** | **.42** | **-2.258** | **0.888** | **.01** | **-0.089** | **0.149** | **.55** | -0.274 | 0.138 | .05 | **-0.089** | **0.147** | **.54** | -0.274 | 0.137 | .05 |
| - 1-2 years | **-0.660** | **2.368** | **.78** | **-6.299** | **1.708** | **<.001** | **0.071** | **0.296** | **.81** | -0.729 | 0.264 | .006 | **0.072** | **0.293** | **.81** | -0.729 | 0.261 | .005 |
| - >2 years | **-5.987** | **2.537** | **.02** | -4.496 | 1.782 | .01 | **-0.786** | **0.321** | **.01** | -0.441 | 0.275 | .11 | **-0.786** | **0.319** | **.01** | -0.441 | 0.272 | .10 |
| Prior treatment |  |  |  |  |  |  |  |  |  |  |  |  |  |  |  |  |  |  |
| - No | -2.985 | 2.220 | .18 | -2.344 | 2.258 | .30 | -0.313 | 0.139 | .02 | -0.200 | 0.133 | .13 | -0.209 | 0.165 | .21 | -0.069 | 0.151 | .65 |
| - Yes | -3.226 | 2.361 | .17 | -4.838 | 2.419 | .05 | -0.411 | 0.176 | .02 | -0.515 | 0.167 | .002 | -0.069 | 0.231 | .77 | -0.248 | 0.209 | .24 |
| Prior episode |  |  |  |  |  |  |  |  |  |  |  |  |  |  |  |  |  |  |
| - No | -2.702 | 1.650 | .10 | -3.967 | 1.922 | .04 | -0.319 | 0.136 | .02 | -0.487 | 0.145 | <.001 | -0.206 | 0.164 | .21 | -0.444 | 0.150 | .003 |
| - Yes | -1.457 | 1.618 | .37 | -3.329 | 1.880 | .08 | -0.234 | 0.127 | .07 | -0.415 | 0.135 | .002 | -0.045 | 0.178 | .80 | -0.229 | 0.164 | .16 |
| History of hospitalization |  |  |  |  |  |  |  |  |  |  |  |  |  |  |  |  |  |  |
| - No | -2.847 | 1.994 | .15 | -3.004 | 2.488 | .23 | -0.307 | 0.126 | .01 | -0.286 | 0.121 | .02 | -0.195 | 0.153 | .20 | -0.143 | 0.139 | .30 |
| - Yes | -6.688 | 2.866 | .02 | -4.979 | 3.322 | .13 | -0.873 | 0.319 | .01 | -0.604 | 0.303 | .05 | 0.558 | 0.672 | .41 | 0.622 | 0.611 | .31 |
| PD comorbidity |  |  |  |  |  |  |  |  |  |  |  |  |  |  |  |  |  |  |
| - No | -0.851 | 1.523 | .58 | -2.457 | 1.565 | .12 | -0.178 | 0.130 | .17 | -0.485 | 0.139 | <.001 | -0.084 | 0.141 | .55 | -0.449 | 0.133 | <.001 |
| - Yes | -0.285 | 1.655 | .86 | -3.863 | 1.745 | .03 | -0.095 | 0.149 | .52 | -0.397 | 0.157 | .01 | -0.171 | 0.163 | .29 | -0.402 | 0.151 | .008 |
| AD comorbidity |  |  |  |  |  |  |  |  |  |  |  |  |  |  |  |  |  |  |
| - No | **-6.249** | **1.251** | **<.001** | **-7.453** | **2.076** | **<.001** | -0.461 | 0.184 | .01 | **-0.959** | **0.181** | **<.001** | -0.078 | 0.246 | .75 | **-1.137** | **0.227** | **<.001** |
| - Yes | **-0.976** | **0.973** | **.32** | **-2.974** | **1.815** | **.10** | -0.177 | 0.143 | .22 | **-0.477** | **0.140** | **<.001** | 0.016 | 0.169 | .92 | **-0.402** | **0.157** | **.01** |
|  |  |  |  |  |  |  |  |  |  |  |  |  |  |  |  |  |  |  |
| Pre-treatment HAMD |  |  |  |  |  |  |  |  |  |  |  |  |  |  |  |  |  |  |
| - Average | -1.716 | 0.493 | <.001 | -3.998 | 0.640 | <.001 | -0.204 | 0.069 | .003 | -0.466 | 0.084 | <.001 | -0.039 | 0.083 | .64 | -0.382 | 0.093 | <.001 |
| - Per point/SD increase† | **-0.396** | **0.048** | **<.001** | **-0.477** | **0.056** | **<.001** | **-0.494** | **0.057** | **<.001** | **-0.598** | **0.066** | **<.001** | **-0.431** | **0.069** | **<.001** | **-0.541** | **0.074** | **<.001** |
| Age |  |  |  |  |  |  |  |  |  |  |  |  |  |  |  |  |  |  |
| - Average | -1.693 | 1.353 | .21 | -3.547 | 1.539 | .02 | -0.260 | 0.096 | .007 | -0.493 | 0.102 | <.001 | -0.120 | 0.122 | .33 | -0.421 | 0.114 | <.001 |
| - Per year increase | -0.077 | 0.050 | .12 | 0.026 | 0.057 | .65 | -0.013 | 0.008 | .10 | 0.002 | 0.008 | .80 | **-0.020** | **0.010** | **.05** | -0.007 | 0.009 | .44 |
| CGI-S |  |  |  |  |  |  |  |  |  |  |  |  |  |  |  |  |  |  |
| - Average | -0.963 | 1.333 | .47 | -2.826 | 1.638 | .08 | -0.117 | 0.121 | .33 | -0.426 | 0.114 | <.001 | -0.121 | 0.121 | .32 | -0.430 | 0.113 | <.001 |
| - Per point increase | 0.563 | 0.782 | .47 | 0.051 | 0.783 | .95 | 0.087 | 0.117 | .46 | 0.093 | 0.112 | .41 | 0.087 | 0.116 | .45 | 0.093 | 0.112 | .41 |
| GAF |  |  |  |  |  |  |  |  |  |  |  |  |  |  |  |  |  |  |
| - Average | -1.211 | 1.676 | .47 | -2.851 | 2.090 | .17 | -0.212 | 0.129 | .10 | -0.230 | 0.155 | .14 | -0.223 | 0.151 | .14 | -0.177 | 0.138 | .20 |
| - Per point increase | 0.069 | 0.056 | .22 | 0.088 | 0.060 | .14 | 0.003 | 0.008 | .71 | 0.007 | 0.009 | .44 | 0.004 | 0.010 | .69 | 0.006 | 0.009 | .50 |
| Comorbid anxiety score |  |  |  |  |  |  |  |  |  |  |  |  |  |  |  |  |  |  |
| - Average | -2.082 | 1.419 | .14 | -3.812 | 1.554 | .01 | -0.267 | 0.102 | .01 | -0.503 | 0.099 | <.001 | -0.140 | 0.118 | .24 | -0.449 | 0.111 | <.001 |
| - Per SD increase | 0.190 | 0.536 | .72 | -0.992 | 0.580 | .09 | -0.026 | 0.083 | .75 | **-0.234** | **0.080** | **.003** | -0.076 | 0.093 | .41 | **-0.258** | **0.088** | **.003** |

Note. * These analyses do not include the study by Lopez Rodriguez et al. (2004). † Per one point increase in baseline HAMD score for sensitivity analysis 1 and per one SD increase in baseline HAMD score for sensitivity analyses 2 and 3.

AD = anxiety disorder; B = mean difference; CGI-S = Clinical Global Impression subscale ‘Severity of Illness’; *d* = Cohen’s *d* effect size; GAF = Global Assessment of Functioning Scale; HAMD = Hamilton Depression Rating Scale; *p* = p-value; PD = personality disorder; SD = standard deviation; SE = standard error.

Negative signs indicate lower depressive symptom levels (i.e., better outcomes) in the combined antidepressants and STPP treatment condition than in the comparison condition. Numbers printed in bold indicate statistically significant time-by-moderator-by-treatment 3-way interactions (*p* <.05). For categorical variables, this indicates a significant difference between the treatment effect in this category and another. For continuous variables, the first effect size (‘Average’) reflects the treatment comparison for participants with baseline scores at the average of the study sample, while the second number (‘Per … increase’) reflects the additional effect for each unit increase in baseline score.
